# Comparative Analysis of E2F Family Member Oncogenic Activity

**DOI:** 10.1371/journal.pone.0000912

**Published:** 2007-09-19

**Authors:** Chunxia Chen, Andrew D. Wells

**Affiliations:** 1 Joseph Stokes, Jr. Research Institute, The Children's Hospital of Philadelphia, Philadelphia, Pennsylvania, United States of America; 2 Department of Pathology and Laboratory Medicine, University of Pennsylvania School of Medicine, Philadelphia, Pennsylvania, United States of America; Centre de Recherche Public-Santé, Luxembourg

## Abstract

The E2F family of transcription factors consists of nine members with both distinct and overlapping functions. These factors are situated downstream of growth factor signaling cascades, where they play a central role in cell growth and proliferation through their ability to regulate genes involved in cell cycle progression. For this reason, it is likely that the members of the E2F family play a critical role during oncogenesis. Consistent with this idea is the observation that some tumors exhibit deregulated expression of E2F proteins. In order to systematically compare the oncogenic capacity of these family members, we stably over-expressed E2F1 through 6 in non-transformed 3T3 fibroblasts and assessed the ability of these transgenic cell lines to grow under conditions of low serum, as well as to form colonies in soft agar. Our results show that these six E2F family members can be divided into three groups that exhibit differential oncogenic capacity. The first group consists of E2F2 and E2F3a, both of which have strong oncogenic capacity. The second group consists of E2F1 and E2F6, which were neutral in our assays when compared to control cells transduced with vector alone. The third group consists of E2F4 and E2F5, which generally act to repress E2F-responsive genes, and in our assays demonstrated a strong capacity to inhibit transformation. Our results imply that the pattern of expression of these six E2F family members in a cell could exert a strong influence over its susceptibility to oncogenic transformation.

## Introduction

The E2F family of transcription factors consists of nine members (E2F1, E2F2, E2F3a, E2F3b, E2F4, E2F5, E2F6, E2F7 and E2F8) with both distinct and overlapping functions (reviewed in [1]–[3]). E2F1–6 form heterodimers with DP proteins to achieve high-affinity DNA binding, while E2F7 and 8 do not require these co-factors to bind to E2F target genes. E2F proteins are situated at the ‘bottom’ of the growth factor signaling cascade where they regulate genes involved in cell cycle progression [4], [5], and can act either as transcriptional activators or repressors depending upon their association with pocket proteins such as pRB [1]. For this reason, it is likely that the members of the E2F family are important regulators of oncogenic transformation.

The transforming potential of E2F1–3 has been reported in various models and cell types, however, a systematic comparison of E2F1–6 members has not been performed. To make a direct comparison of oncogenic function among these first six E2F family members, we have utilized a retroviral approach to generate stable lines of 3T3 fibroblasts specifically over-expressing E2F1, E2F2, E2F3a, E2F4, E2F5 or E2F6, and have assessed the ability of these transgenic cell lines to grow under conditions of low serum, as well as to form colonies when suspended in soft agar. Our data demonstrates that E2F2 and E2F3 have strong pro-oncogenic capacity, whereas E2F4 and E2F5 are anti-oncogenic.

## Results

### Generation of 3T3 fibroblast lines over-expressing individual E2F family members

**Figure 1:**
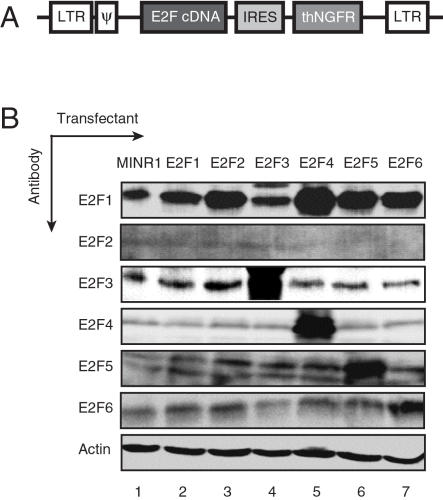
Generation of retroviral vectors encoding E2F family members. A. Schematic representation of MINR1 retroviral vectors encoding E2F genes. B. Analysis of E2F family member expression in transiently-transfected 293T cells. 293T cells were individually transfected with empty (lane 1) or E2F1–6 retroviral plasmids (lanes 2–7), and extracts were subjected to SDS-PAGE, blotted, and probed for individual E2F1–6 (top six panels) or actin (bottom panel).

To achieve stable, forced expression of E2F family members 1 through 6 in cells, we constructed bicistronic retroviral vectors encoding E2F1, E2F2, E2F3a, E2F4, E2F5 and E2F6 (Figure 1 A). These constructs were able to drive high-level expression of the NGFR reporter protein (data not shown), as well as specific over-expression of E2F3a, E2F4, E2F5 and E2F6 protein, respectively, upon transient transfection of Pheonix/293T cells (Figure 1 B). E2F1 over-expression was only variably achieved under these conditions, potentially due to high basal expression of endogenous E2F1 by Pheonix cells (Figure 1 B, top panel). We also had difficulty demonstrating E2F2 over-expression in these transient transfections, either due to low level expression of E2F2 protein, or relatively low sensitivity of the E2F2-specific antiserum (Figure 1 B, second panel).

**Figure 2:**
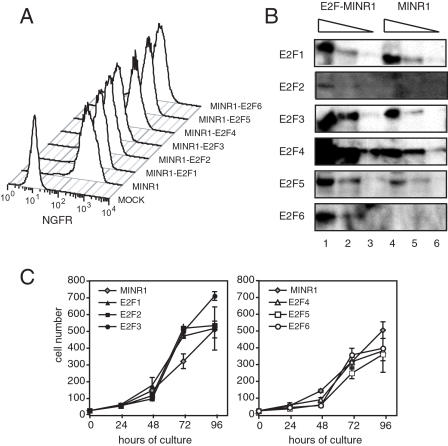
Effect of forced E2F expression on exponential growth of NIH-3T3 cells. A. NIH-3T3 cells were transduced with retroviral constructs encoding individual E2F1–6, and stained for surface expression of human mutant NGFR. B. Serial 3-fold extracts of stable empty MINR1 (lanes 4–6) or MINR1-E2F1–6 (lanes 1–3) 3T3 transductants were analyzed for E2F expression as in Figure 1. C. 2×10^5^ of each transductant (E2F1–3, left panel; E2F4–6, right panel) was seeded and cultured in full medium with 10% serum. Cultures were counted every 24 hours, and are in thousands. Data are representative of 2 separate experiments.

E2F-encoding retroviral supernatants produced from these Pheonix cell transfections were used to transduce non-transformed NIH 3T3 fibroblasts, and transductants were identified and purified by the expression of NGFR (Figure 2 A). Transduced 3T3 lines were expanded without selection, and stable NGFR expression was observed over several weeks in culture (data not shown). We were able to detect specific over-expression of E2F2 through E2F6 in each respective 3T3 line under conditions of asynchronous growth, as compared to endogenous expression of these family members in an empty vector-transduced line (Figure 2 B). However, we were unable to detect over-expression of E2F1 in actively growing, E2F1-transduced 3T3 cells above that of the endogenous protein (Figure 2 B).

### Effect of deregulated E2F expression on asynchronous cell growth

To determine the effect of stable over-expression of individual E2F family members on asynchronous cell growth, we plated each E2F-transduced line at low density in high (10%) serum medium, and enumerated the cells at 24 hour intervals over a four day culture period. The empty vector-transduced 3T3 line exhibited a consistent doubling rate of approximately 24 hours until reaching confluency between 72 and 96 hours (Figure 2 C, gray diamonds). This pattern of growth closely resembled that of the parental, non-transduced 3T3 cells (data not shown). The E2F1-, E2F2-, and E2F3-transduced lines exhibited a normal growth rate during the first 48 hours of culture, but then proliferated at twice the rate of the control cells until reaching confluency after only 72 hours (Figure 2 C, filled symbols). Over the next 24 hours, the E2F1- and E2F2-transduced lines underwent growth arrest (Figure 2 C, filled squares and triangles), suggesting that these lines are still susceptible to contact inhibition. However, the E2F3-transduced line continued to grow at the same rate 24 hours after reaching confluency (Figure 2 C, filled circles), suggesting that forced expression of E2F3 can overcome contact inhibition. Unlike the E2F1–3 lines, cells transduced with E2F4, E2F5 and E2F6 lagged behind the control MINR1 3T3 line, exhibiting little or no cell growth over the first 48 hours of culture (Figure 2 C, open symbols). These lines underwent approximately two doublings during the next 24 hours, but arrested at roughly 72 hours, before reaching 100% confluence. These data suggest that deregulated expression of E2F4, E2F5 and E2F6 can slow cell cycle progression and render cells more susceptible to contact inhibition.

### Effect of deregulated E2F expression on serum-independent cell growth

**Figure 3:**
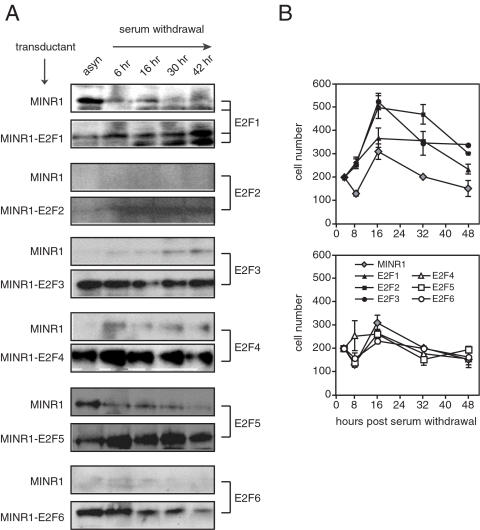
Capacity of individual E2F family members to support serum-independent growth. 2×10^5^ asynchronous 3T3 transductants from exponential cultures were seeded overnight with 10% serum, then cultured in 0.1% serum for the indicated periods, and E2F protein levels (A) and live cell counts (B) were measured. The data depicted are representative of 2 separate experiments.

E2F gene expression is normally regulated by signals from growth factor receptors, and is tightly coordinated with the cell cycle. To determine the effect of forced expression of individual E2F family members on cell growth in the relative absence of growth factors, we plated each E2F-transduced line at medium density in low (0.1%) serum medium, and monitored E2F family member expression and cell number over a two day culture period. The MINR1 empty vector line exhibited very low expression of endogenous E2F family members following serum withdrawal (Figure 3 A), while the E2F3–6 transductants exhibited efficient, serum-independent expression of E2F3, E2F4, E2F5 and E2F6, respectively (Figure 3 A). Serum deprivation actually induced accumulation of transgenic E2F1 and E2F2 protein in the E2F1- and E2F2-transduced lines (Figure 3 A), suggesting that growth factor-coupled mechanisms that limit E2F1 and E2F2 protein expression at a post-translational level may be operative during growth in high serum in our system [6], [7].

During the first 16 hours of serum deprivation, control-transduced 3T3 cultures increased in cell number by approximately 50%, but fell precipitously by 30 hours (Figure 3 B, gray diamonds). These results, along with the initial drop in cell number at 8 hours, suggest a relatively rapid conversion from cell growth to cell death in these cultures upon serum withdrawal. Conversely, 3T3 cells with forced expression of E2F1, E2F2 and E2F3 continued to grow following serum deprivation (Figure 3 B, filled symbols). E2F2- and E2F3-transduced cultures continued to double after 16 hours of serum withdrawal, and maintained at least two-fold greater cell numbers than control cultures throughout the 48 hour culture period (Figure 3 B, filled squares and circles). E2F1-transduced cultures showed a more modest rate of growth, however, these cultures were also able to maintain significantly increased cell numbers throughout serum deprivation as compared to empty vector-transduced cultures (Figure 3 B, filled triangles). Unlike the lines with forced expression of E2F1, 2 or 3, cell lines transduced with E2F4, E2F5 and E2F6 did not continue to grow following serum withdrawal, and maintained cell numbers equal to or less than the control-transduced cultures throughout the entire response (Figure 3 B, open symbols). These data show that uncoupling of E2F1, E2F2 and E2F3 expression from their normal growth factor-mediated regulation is sufficient to drive a significant degree of growth factor-independent cell cycle progression, but that E2F4, 5 and 6 cannot mediate this effect under the same conditions.

### Effect of deregulated E2F expression on anchorage-independent cell growth

**Figure 4:**
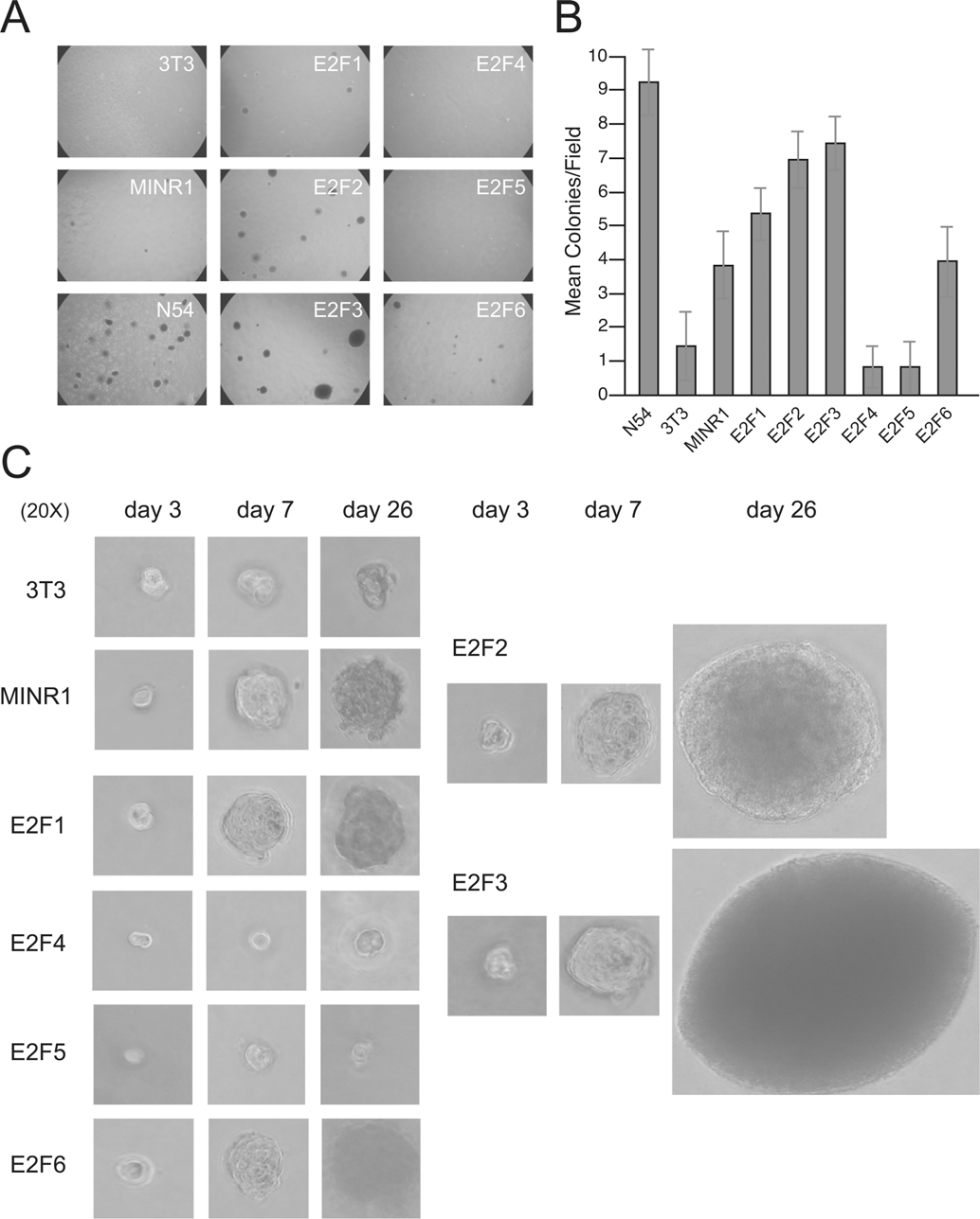
Capacity of individual E2F family members to support contact-independent growth. 10^3^ E2F-3T3 transductants were seeded in soft agar medium with 10% serum. Representative 4× fields of N54 (Ras-transformed positive control), empty vector, and E2F1–6 transductant cultures at 30 days are shown in A. The mean colony number per field (+/− SD) from 10 random fields is plotted in B. The largest colony from each transductant culture at days 3, 7 and 26 is depicted (20×) in C. The data depicted in A–C are representative of 4 separate experiments.

The growth of most cells, including fibroblasts, requires integrin-mediated signals provided through attachment to a solid matrix. One characteristic of cancer cells is the loss of this requirement, and such transformed cells gain the capacity to grow in an anchorage-independent manner [8]. To simulate these conditions and assess this oncogenic characteristic, we cultured stable E2F-expressing 3T3 lines in suspension in a semi-solid agarose medium. In this system, the non-transformed parental 3T3 cell line exhibited an almost complete requirement for attachment, as only a few small colonies were observed which did not show continuous growth when cultures were extended up to two months (Figure 4 A–C). Positive control H-Ras-transformed N57 cells generated a high frequency of large colonies that grew progressively over a one month period (Figure 4 A–C). The empty MINR1 vector-transduced 3T3 line showed a significant increase in the frequency of colony formation as compared to the parental line (Figure 4 A and B), but unlike the progressive growth of the N57 colonies, the MINR1-3T3 colonies were small, and had involuted by day 20 (Figure 4 C). Ectopic expression of E2F1 and E2F6 in 3T3 cells had a relatively neutral effect on colony formation as compared to the empty vector-transduced line (Figure 4 A–C). In contrast, the E2F2- and E2F3-transduced 3T3 lines exhibited strong colony forming capacity. These cells generated colonies at frequencies approaching the H-Ras-transformed N57 cells (Figure 4 A and B), with individual colonies exhibiting strong, exponential growth over the entire one month culture period (Figure 4 C). Interestingly, ectopic expression of E2F4 and E2F5 in 3T3 fibroblasts resulted in frequencies of colony formation that were significantly lower than that of empty vector-transduced cells, and comparable to that of the non-transformed parental 3T3 line (Figure 4 A and B). Furthermore, the few colonies present in these cultures did not grow over time (Figure 4 C). These data suggest that E2F2 and E2F3 have strong oncogenic capacity, while E2F4 and E2F5 are anti-oncogenic in this system. E2F1 and E2F6 may be weakly oncogenic, but the background transforming capacity of the retroviral vector used in these studies makes our results with these two family members difficult to interpret.

## Discussion

A number of genetic aberrations that promote cancer lead to deregulated E2F activity [9], including mutations in pRb, cyclinD1, p16INK4a and CDK4. Although E2F genes are not frequent targets of mutations in cancer [10], amplification and/or dysregulation of E2F expression is associated with malignancy in several tumors [11]–[13]. By forcing stable, homogeneous expression of individual E2F family members in non-transformed parental cells, our approach provides a model of dysregulated E2F expression, and allows an unprecedented systematic comparison of the oncogenic capacity of six different E2F family members. Our results show that these six E2F family members have very different effects on cell growth under conditions of limiting mitogenic signals.

In our studies, retroviral expression of E2F2 and E2F3 promoted both serum- and contact-independent growth of normal fibroblasts, consistent with previous *in vitro* studies in both transient and stable over-expression systems [14], [15]. These data are also consistent with *in vivo* studies in which targeted expression of E2F2 or E2F3 in epithelial tissue led to epithelial hyperplasia, and in the case of deregulated E2F2 expression, led to cortical thymoma formation [16], [17]. In our studies, E2F3a exhibited stronger transformation activity than E2F2. This may result from the more stable expression of transgenic E2F3a protein in this system as compared to E2F2 (Figures 2 and 3), and suggests that E2F3a and E2F2 may be differentially subject to post-translation control mechanisms [6]. Also, while E2F1, E2F2 and E2F3a can each contribute to the initial G0-S phase progression following stimulation of quiescent cells, E2F3a is the predominant family member involved in subsequent G1-to-S phase transitions [18], and has a unique role in centrosome duplication [19]. These activities may together account for strongest proliferative capacity of the E2F3a-transgenic fibroblasts in our studies. E2F3b, a splice variant of E2F3 that contains coding regions unique from E2F3a [20], was not tested in these studies. This family member might be expected to be neutral or anti-oncogenic, as E2F3b has been shown to preferentially bind pRb and repress S-phase genes in fibroblasts *in vitro* [21], but further studies will be required to address the oncogenic capacity of this E2F family member.

Forced expression of E2F4 and E2F5 negatively impacted fibroblast growth in our experiments, consistent with their defined roles in enforcing G1 arrest [22]. E2F4 and E2F5 can exhibit oncogenic activity, but only when expressed together with other oncogenes such an activated mutant of Ras [11], [23]. The empty MSCV retroviral vector in our studies exhibited measurable transforming activity in 3T3 fibroblasts, and this was abrogated by E2F4 and E2F5. These results suggest that these E2F family members can also have anti-oncogenic or tumor suppressive activity. Unlike E2F1–3, E2F4 and E2F5 are highly expressed in quiescent (G0) cells, lack a cyclin A-binding domain, and associate with p107 and p130 instead of pRB. These factors also lack nuclear localization domains, and depend upon their association with pocket proteins for nuclear translocation [23]–[26]. Consequently, E2F4 and E2F5 commonly act as repressors of E2F responsive genes [22], which may explain why forced expression of these factors inhibits proliferation and transformation in our studies. Two new members of an E2F subfamily, E2F7 and E2F8, were recently identified after our studies were performed, and like E2F4 and 5, act as repressors of E2F-induced gene expression and mitotic progression [27]–[30]. For this reason we would predict that these new factors would have anti-oncogenic properties, however, further studies will be required to address this issue.

E2F1 and E2F6 had weak or no oncogenic capacity compared to empty vector-transduced cells in our system. E2F6 is unique in that it retains the conserved E2F DNA binding and dimerization domains, but lacks the C-terminal transactivation and pocket protein binding domains characteristic of other members [31]–[33]. Therefore, E2F6 can act as a competitive inhibitor of DNA binding by other E2F proteins, and when overexpressed can oppose the function of both the oncogenic E2F2 and 3a proteins and the anti-oncogenic E2F4 and 5 family members. This behavior may explain why E2F6 is neutral in our transformation assays. E2F6 may also repress pro-mitogenic E2F-responsive genes, as the C-terminal portion of E2F6 encompassing the marked-box domain has been shown to inhibit gene transcription through the recruitment of co-repressor complexes [22], [34], [35]. This scenario is supported by our *in vitro* data, in which forced expression of E2F6 delayed serum-induced cell growth.

In our experiments, forced expression of E2F1 could support serum-independent growth, which is consistent with previous studies [36]. Dysregulated E2F1 expression can promote hepatocellular adenoma [37], spontaneous epithelial tumors [38], or in combination with activated ras or p53 deficiency, accelerate skin tumorigenesis [39], [40]. However, this factor was significantly less efficient in promoting *in vitro* growth than E2F2 or E2F3 (Figures 2 and 3), and in our soft agar culture system E2F1 exhibited very weak colony forming activity over control-transduced 3T3 fibroblasts. This weak oncogenic activity could result from post-translational destabilization of E2F1 through ubiquitination [7], and indeed we found that E2F1 protein was expressed less efficiently from the same vector as compared to E2F3 (Figure 3). These results contrast two previous studies, which showed that stable over-expression of E2F1 in fibroblasts could induce measurable contact-independent cell growth [15], [41]. One of these studies generated stably-transfected rat embryonic fibroblast lines through drug selection, and achieved very high levels of E2F1 expression [41]. The other study utilized a MoMuLV-based vector [15], which may contribute less background transforming activity than our MSCV-based vector in these studies, and therefore may allow detection of weaker oncogenes. In the majority of previous studies, however, E2F1 activity has been shown to oppose proliferation and oncogenesis [42], [43] through its strong capacity to activate the p53/73 pathway of intrinsic cell death [44], [45], which likely acts to balance its pro-mitogenic activity in these assays. Whether the balance of E2F1 activity in a specific tissue leads to apoptosis and tumor suppression vs. proliferation and oncogenesis is likely dependent upon the context of pro- vs. anti-apoptotic signals received by cells at a given time.

In this study, we systematically compared the transforming activity of E2F family members 1 through 6. Our results show that these six E2F family members can be divided into three groups based upon their oncogenic capacity in fibroblasts: 1) strong oncogenes (E2F2 and E2F3a), 2) weak or neutral genes (E2F1 and E2F6), and 3) anti-oncogenes (E2F4 and E2F5). The differential capacity of these E2F factors to promote oncogenic cell growth was associated with their protein stability, and is likely influenced by their normal expression patterns and cooperation with other factors.

## Materials and Methods

### Construction of retroviral vectors encoding E2F proteins

The plasmids pcDNA1-mE2F1 and pBS-mE2F3a were provided by J. Nevins (Duke Univ.), and plasmids pCMVHA-hE2F2, pCMVHA-hE2F5, pCMVHA-hE2F6 were provided by K. Helin (Eur. Inst. Oncology). Full-length E2F cDNA fragments were subcloned into the EcoRV site of pST-Blue1 vector (Novagene) by blunt-end ligation. Murine E2F4 cDNA was cloned by RT-PCR from NIH 3T3 cell lines. Briefly, total RNA was prepared from 10^7^ NIH 3T3 cells in RNAstat60 (Tel-test), precipitated in isopropanol, and dissolved in DEPC-treated water. First strand cDNA synthesis from 2 µg of total RNA was achieved using random hexamers and super-reverse transcriptase (Invitrogen). One-tenth of the RT product was used for PCR using the following primers: mE2F4 forward primer; 5′-CCG GAA TTC CGG GAT GGC GGA GGC CGG GCC ACA GG-3′, mE2F4 reverse primer; 5′-CCG GAA TTC CGG GGG TTG CAG CTG CAC AGG ACA TG-3′. The PCR product was cloned into the EcoRI site of pST-Blue1 vector (Novagen), and confirmed by sequencing. All E2F cDNAs were subcloned from pST-Blue1 into the EcoRI cloning site of the MSCV-NGFR expression vector (MINR1, provided by W. Pear, Univ. Penn.). In this vector, each E2F ORF is expressed as a bi-cistronic mRNA linked to a truncated human nerve growth factor receptor (NGFR) reporter gene by an IRES element, and transduced cells can be identified by surface expression of NGFR [46]. The general structure of these vectors is shown in Figure 1 A. All MINR1-E2F inserts were sequenced using MINR1-specific forward and reverse primers (s5: 5′-CCT CCG CCT CCT CTT CCT CCA TCC-3′ and a6: 5′-GCC AAA AGA CGG CAA TAT GGT GG-3′).

### Cell lines

The 293T-based Pheonix ecotropic packaging cell line (provided by G. Nolan, Stanford Univ.) was used for retroviral vector production. Gag-pol and env expression was ensured by selection in medium containing hygromycin and diptheria toxin every three months. NIH 3T3 cells (ATCC) were maintained in DMEM medium supplemented with 10% FBS, in 37C and 5% CO2 and used for E2F cell line generation.

### Retrovirus production

Pheonix cells were transiently transfected with MINR1-E2F retroviral constructs using Lipofectamine 2000 reagent (Invitrogen) as described by the manufacturer. Briefly, highly confluent Pheonix cells were co-transfected with a mixture of X µg MINR1 and X µg pCLeco (Invitrogen) plasmid DNA mixed with Lipofectamine 2000 for 4–6 hours. The transfection mixture was then replaced with fresh growth medium and the cells were cultured for 48 hours. The retroviral supernatants were passed through 0.45 µm filter, aliquoted and stored at −70C for future use.

### NIH/3T3 cell transductions

NIH 3T3 cells (3×10^4^) were seeded in 24 well plates, cultured overnight, and incubated with 1 mL retroviral supernatant in the presence of 8 µg/ml polybrene (Sigma) at 37C for 18 hours. The virus-containing medium was replaced with fresh medium and cells were cultured for an additional 48 hours.

### Flow cytometry

Transfections and transductions were monitored by flow cytometry by surface staining for the hNGFR reporter gene product. Briefly, Pheonix cells or 3T3 cells were harvested by trypsinization, washed in PBS with 1% horse serum, and 5×10^5^ cells were stained with anti-NGFR-Biotin antibody (BD-Biosciences) at 4C for 30 min. Cells were washed and stained with streptavidin-PE (BD-Biosciences) at 4C for 30 min. Cells were analyzed on Cyan flow cytometer (Dako).

### Serum deprivation and serum stimulation

E2F-3T3 transductants were plated in triplicate at 2×10^6^ cells in 10 cm dishes. After 24 hours, the cells were washed twice with PBS and then incubated in DMEM containing 0.1% FBS for 72hr. The cells were then fed with DMEM containing 20% FBS. At each time point, the cells were harvested by trypsin treatment and counted using a hemocytometer.

### Immunoblot analysis

E2F-3T3 cells (2×10^5^) were boiled in reducing Laemmli buffer and subjected to SDS-PAGE. Cellular proteins were transferred to nitrocellulose membranes and probed with specific antisera specific for E2F1, E2F2, E2F3a/b, E2F4, E2F5 and E2F6 (Santa Cruz). Immunoreactive proteins were detected with HRP-conjugated secondary antibody (Jackson Immunoresearch) and visualized using chemiluminescence (BioRad).

### Semi-solid agar culture of E2F-3T3 cell lines

Actively growing 3T3 cells (1×10^3^) were mixed with pre-warmed medium supplemented with 0.3% agarose (type VII, Sigma), plated onto solidified medium containing 0.5% agarose in 6-well plates, and cultured at 37C in 5% CO2. Top agarose was replenished every two weeks. Colony formation was monitored and enumerated by counting 10 random 10× fields.
